# Functional Characterization and Structural Analysis of NADH Oxidase Mutants from *Thermus thermophilus* HB27: Role of Residues 166, 174, and 194 in the Catalytic Properties and Thermostability

**DOI:** 10.3390/microorganisms7110515

**Published:** 2019-10-31

**Authors:** Javier Rocha-Martin, Pedro A. Sánchez-Murcia, Fernando López-Gallego, Aurelio Hidalgo, José Berenguer, José M. Guisan

**Affiliations:** 1 Department of Biocatalysis, Institute of Catalysis and Petrochemistry (ICP) CSIC, Campus UAM, Cantoblanco, 28049 Madrid, Spain; 2 Institute of Theoretical Chemistry, Faculty of Chemistry, University of Vienna, Währinger Str. 17 A-1090 Vienna, Austria; pedro.murcia@univie.ac.at; 3 Heterogeneous Biocatalysis Laboratory. Instituto de Síntesis Química y Catálisis Homogénea (ISQCH-CSIC), University of Zaragoza, 50009 Zaragoza, Spain; flopez@cicbiomagune.es; 4 ARAID, Aragon I+D Foundation, Av. de Ranillas 1-D, planta 2^a^, oficina B, 50018 Zaragoza, Spain; 5 Department of Molecular Biology, Universidad Autónoma de Madrid, Center for Molecular Biology Severo-Ochoa (UAM-CSIC), Nicolás Cabrera 1, 28049 Madrid, Spain; ahidalgo@cbm.csic.es

**Keywords:** NADH oxidase, cofactor regeneration, hydrogen peroxide, dehydrogenase, NAD^+^, extremophiles

## Abstract

The *Thermus thermophilus* strain HB27 NADH-oxidase (Tt27-NOX) catalyzes the oxidation of nicotinamide adenine dinucleotide (NAD(P)H) by reducing molecular oxygen to hydrogen peroxide in a two-electron transfer mechanism. Surprisingly, Tt27-NOX showed significant differences in catalytic properties compared to its counterpart from the strain HB8 (Tt8-NOX), despite a high degree of sequence homology between both variants. The sequence comparison between both enzymes revealed only three divergent amino acid residues at positions 166, 174, and 194. Motivated with these findings, in this work we performed mutagenesis experiments in the former three positions to study the specific role of these residues in the catalytic properties and thermostability of Tt27-NOX. We subjected five mutants, along with the wild-type enzyme, to biochemical characterization and thermal stability studies. As a result, we identified two more active and more thermostable variants than any Tt8-NOX variant reported in the literature. The most active and thermostable variant K166/H174/Y194 retained 90% of its initial activity after 5 h at pH 7 and 80 °C and an increase in melting temperature of 48.3 °C compared with the least active variant K166/R174/Y194 (inactivated after 15 min of incubation). These results, supported by structural analysis and molecular dynamics simulation studies, suggest that Lys at position 166 may stabilize the loop in which His174 is located, increasing thermal stability.

## 1. Introduction

*Thermus thermophilus* HB27 is a thermophilic bacterium belonging to a phylogenetically ancestral phylum [1] used as laboratory model for thermophiles [2]. It grows up fast to reach high cell densities at 72 °C with oxygen as an electron acceptor. Among its thermophilic oxygen reacting proteins, the NADH oxidase (Tt-NOX) has been well characterized despite its actual physiological function in vivo not being known [3,4].

Tt-NOX (EC 1.6.3.3) is a homodimeric flavoprotein (monomer *M*_r_ ~27 kDa) which belongs to the flavin reductase and nitroreductase superfamily [4,5]. This enzyme catalyzes the oxidation of reduced nicotinamide adenine dinucleotide (NAD(P)H) by reducing molecular oxygen to hydrogen peroxide in a two-electron transfer mechanism [3]. Each monomer can accept one molecule of flavin mononucleotide (FMN) or flavin adenine dinucleotide (FAD) as a cofactor. Besides molecular oxygen, FAD, ferricyanide, cytochrome c, methylene blue, and others can act as an electron acceptor [4]. The three-dimensional crystal structure of the NADH oxidase isolated from the *T. thermophilus* strain HB8 (Tt8-NOX) is available (PDB id. 1NOX, doi: 10.2210/pdb1nox/pdb). The active site of NADH oxidase (NOX) is located at the edge of the dimeric interface along a large contacting area stabilizing the intermonomeric cleft [4]. Previous studies have shown that the activation of the NOX is caused by the increase in the dynamics of the polypeptide and side chains in the enzyme active site produced by high-temperatures, anions and urea among others [6,7,8,9]. Molecular dynamic (MD) studies have proved the existence of two different conformations of the active site in a dynamic equilibrium: Open and closed conformations [5,9]. Trp 47 has been described as the key residue responsible for controlling the accessibility of the flavin ring, probably playing a critical role throughout the catalytic process [5].

NOXs from thermophile microorganisms are of special interest for the development of biosensors and cofactor recycling systems due to their high thermostability, which will extend the useful lifetime of these systems. Besides, the high-temperature resistance simplifies their purification by heat treatment when the enzymes are overproduced in mesophilic hosts [10]. Tt-NOX presents high chemical and thermal stability, broad-range pH activity, and it can accept NADH or NADPH as substrates [3,10]. Thus, Tt-NOX has successfully been applied to cofactor regeneration systems in the kinetic resolution of secondary alcohols [10] and the selective oxidation of glycerol to yield 1,3-dihydroxyacetone [11,12], in the in situ production of H_2_O_2_ coupled to immobilized preparation of peroxidase from horseradish to oxidize pollutants in aqueous solutions [13], in the activation of Pt^IV^ and Ru^II^ chemotherapeutic agents [14], and in biosensors [15,16,17,18,19,20].

**Figure 1:**
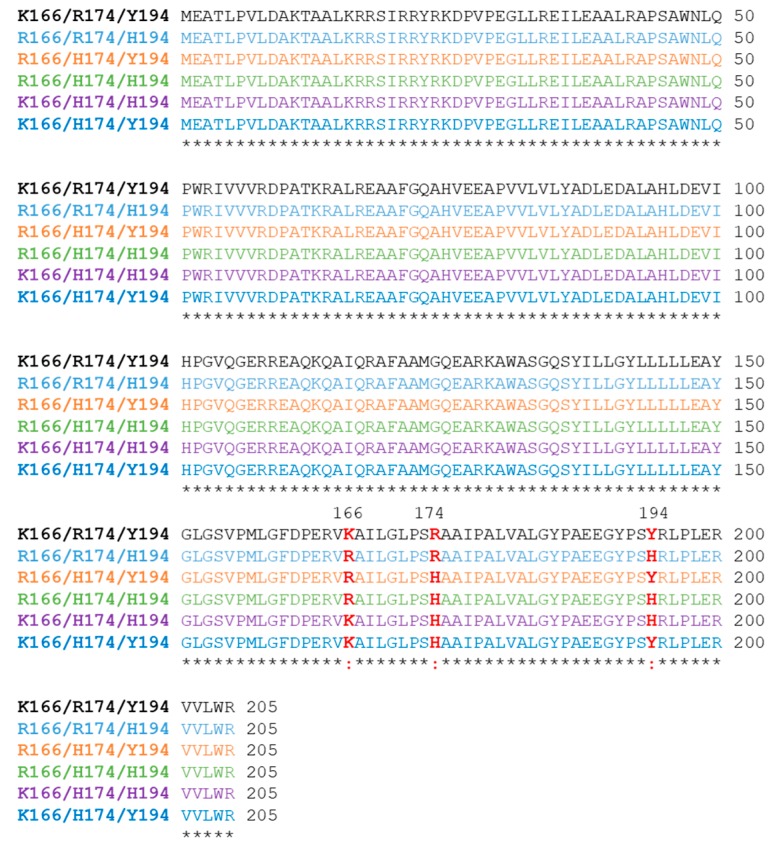
Sequence alignment of the different constructed variants of NOX. Sequence alignment was performed using the ClustalW algorithm. (*) Identical residues; (:) different residues, highlighted in red. K166/H174/Y194 variant sequence resulted from cloning and sequencing of PCR product amplified from genomic DNA of *T. thermophilus* HB27 that we have at our laboratory. K166/H174/H194 variant: the sequence of the variant as it appears in Kyoto Encyclopedia of Genes and Genomes (KEGG, available online: http://www.genome.jp/kegg/kegg2.html). R164/R174/H194 variant sequence corresponds to gene bank accession number CAA42707.1, 1NOX pdb code (doi: 10.2210/pdb1nox/pdb) and it is the variant from *T. thermophilus* HB8 described by Park et al. [3]. R166/H174/H194 variant sequence corresponds to the sequence as it appears in the BacMap genome atlas (Available online: http://wishart.biology.ualberta.ca/BacMap/). K166/R174/Y194 and R166/H174/Y194 variants correspond to the other two mutants studied in this work.

In a previous paper, our research group purified and biochemically characterized an NADH oxidase isolated from the *T. thermophilus* strain HB27 (Tt27-NOX) [10]. In addition, we designed, developed, and characterized a high-stable heterogeneous biocatalyst, which was used for cofactor recycling. The analysis of the Tt27-NOX amino acid sequence revealed a 98.5% identity to a similar enzyme isolated from the HB8 strain. Three divergent amino acid residues were identified at the positions 166, 174, and 194 (Figure 1). Despite a high degree of sequence homology between both variants, we observed differences in their catalytic efficiency [10]. Furthermore, Tt27-NOX variant differed from the sequence of the strain HB27 published by Henne et al. [21] where a single amino acid changes at positions 194 (H194Y) (Figure 1). Therefore, this result opened an opportunity to further optimize the catalytic properties of the Tt-NOX through protein engineering.

In this paper, we performed mutagenesis experiments at the positions 166, 174, and 194 to shed light on the specific role of these three residues that differ between both proteins, on their catalytic properties. Five NOX mutants were constructed, cloned into a pET22b expression vector, overexpressed in *Escherichia coli* BL21, and compared to the variant described by our group (K166/H174/Y194) in a previous study [10]. Once the six variants were purified, the thermal stability and biochemical features were studied. To better understand the differences found between the six NOX variants, structural analysis was also developed, mainly focusing on the most active and thermostable variant (K166/H174/Y194) compared to the most unstable and least active variant (K166/R174/Y194).

## 2. Materials and Methods

### 2.1. Materials

Nicotinamide adenine dinucleotide (NADH) was purchased from Gerbu Biotechnik GmbH (Wieblingen, Germany). Flavin adenine dinucleotide (FAD), flavin mononucleotide (FMN), riboflavin, polyethyleneimine (PEI) (MW: 600–1000 kDa), and sulfate-dextran (MW: 100 kDa) were supplied by Sigma-Aldrich Co (St. Louis, IL, USA). Iminodiacetic acid disodium salt monohydrate (IDA) and copper sulphate (II) 5-hydrate were purchased from Fluka (Buchs, Switzerland). Cyanogen bromide 4B Sepharose (Ag-CB) was from GE Healthcare (Uppsala, Sweden). Cross-linked agarose beads (4%) (BCL) were from Agarose Beads Technology (Madrid, Spain). Agarose coated with polyethyleneimine (PEI-Ag) supports were prepared as previously described elsewhere [19]. Agarose coated with sulfate-dextran agarose beads (DS-Ag) were prepared as previously described [20]. Protein concentration was determined using the method of Bradford [21]. All other used reagents were of analytical grade.

### 2.2. Cloning and Expression of the Tt-NOX Variants

#### 2.2.1. Bacterial Strain and Growth Conditions

The *T. thermophilus* HB27 (DSM 7039) used as a DNA source of the Tt-NOX of the code TTC0057 was a laboratory-adapted strain derived from the original strain donated by Prof Koyama [22]. *E. coli* strains DH5α [*sup*E44, ∆*lac*U169 (∆80 *lac*ZDM15), *hsd*R17, *rec*A, *end*A1, *gyr*A96, *thi-1, relA1*] and BL21 DE3 [*hsd*S, *gal* (∆*c*Its857, *ind*1, *Sam7, nin5, lacUV5*-T7 gene 1)] were used for cloning and expression purposes, respectively. *T. thermophilus* was grown at 70 °C in *Thermus* broth [23] under stirring (150 rpm) and *E. coli* was grown at 37 °C in modified Luria-Bertani (LB) medium [24]. For plasmid selection, growth media was supplemented with kanamycin (30 mg/L) or ampicillin (100 mg/mL). DNA isolation, plasmid purification, restriction analysis, plasmid construction, and DNA sequencing were carried out by standard methods [25].

#### 2.2.2. Site-directed Mutagenesis to Create Tt-NOX Variants

To overexpress the Tt27-NOX, we used the plasmid pET22b-TTC0057 (pET22b-K166/H174/Y194) described previously [10]. A site-directed mutagenesis protocol was used to construct five Tt27-NOX mutants (K166/R174/Y194, K166/H174/H194, R166/H174/Y194, R166/H174/H194, and R166/R174/H194). K166/R174/Y194 (replacing H/R in NOX position 174), K166/H174/H194 (replacing Y/H in NOX position 194) and R166/H174/Y194 (replacing K/R in NOX position 166) mutants were made using the corresponding pair of oligonucleotides (Table S1) as a primer pair in a PCR using the native pET22b-K166/H174/Y194 plasmid as a template and a mixture of *Tth* and *Pfu* DNA polymerases. The product of the PCR was digested with *Dpn*I that exclusively restricts methylated DNA. *E. coli* DH5α cells were transformed directly with the digested product. The plasmids bearing the mutated NOX genes were identified by sequencing and transformed into *E. coli* BL21(DE3) to express the corresponding proteins. Plasmids products are listed in Table S2.

For the second round of mutagenesis, plasmid DNA pET22b-K166/H174/H194 was used as a template and K166R-F/K166R-R as a pair of primers (Table S1). This construction (pET22b-R166/H174/H194) was used as a template for the third round of mutagenesis, following the same procedure as before and using a H174R-F/H174R-R pair of primers (Table S1). The resulting construction was named pET22b-R166/R174/H194 (K166R, H174R, and Y194H; Table S2).

### 2.3. Expression of the Recombinant NOX Variants in E. coli

*E. coli* BL21 (DE3) cells were transformed with the recombinant plasmid pET22b, which carries the RNA polymerase gene from the T7 phage under the control of an inducible promoter. The transformed cells were grown at 37 °C in of LB with ampicillin until the culture reached an optical density of 0.6 at 600 nm. Then, the expression of the T7 RNA polymerase was induced by addition of isopropyl-1-thio-β-d-galactopyranoside (IPTG) to a concentration of 1 mM. The bacterial culture was incubated at 37 °C for further 2 h, and cells were harvested and washed in sodium phosphate buffer by centrifugation (10,000× *g*, 10 min) before being stored as wet pellets at −20 °C until use.

### 2.4. Purification of the Tt-NOX Variants

Cells were lysed by sonication, and the cell debris was eliminated by centrifugation (10,000× *g* for 15 min). Crude protein extracts were diluted 10-fold in 25 mM sodium phosphate pH 7. K166/H174/Y194, K166/H174/H194, and R166/H174/H194 variants were incubated at 80 °C and pH 7 for 45 min. In the case of R166/H174/Y194 and R166/R174/H194 variants, they were incubated at 70 °C and pH 7 for 1 h. Protein aggregates were discarded after centrifugation (10,000× *g* for 10 min), and the clarified supernatant containing the Tt-NOX activity was offered to PEI-Ag and DS-Ag ion-exchange supports at pH 7 and 25 °C (1 g of support per 10 mL of protein extract). K166/R174/Y194 variant was incubated at 70 °C and pH 7 for 1 h. After centrifugation, the clarified supernatant was offered to PEI-Ag at pH 7 and 25 °C (1 g of support per 10 mL of protein extract). Periodically, NOX activity and protein concentration were analyzed in both the supernatant and the suspension fractions to monitor the purification process. Finally, supernatants were analyzed by SDS-PAGE [26].

### 2.5. Determination of Enzyme Activity and Kinetics Parameters

Enzymatic assays were carried out in a spectrophotometer with a thermostated cell and continuous magnetic stirring (Jasco V-630). The activities of the different Tt-NOX preparations were analyzed by following the decrease in absorbance at 340 nm corresponding to the oxidation of NADH. A sample of the enzyme preparation (10–100 µL) was added to a spectrophotometer cuvette containing 2 mL of 50 mM sodium phosphate buffer pH 7, 50 µL of 10 mM NADH, and the indicated concentration of FAD, FMN, or riboflavin. One activity unit (U) was defined as the amount of enzyme required to oxidize 1 micromol of NADH per minute under given conditions and on the basis of the ε = 6.22 mM^−1^·cm^−1^ for NADH at 340 nm.

Kinetic parameters were calculated by measuring the initial velocity of NADH consumption assays [27]. Steady-state kinetic parameters were calculated towards different concentrations of NADH (0–100 µM) with a fixed concentration of FAD (50 µM). Kinetic parameters for riboflavin (0–250 µM), FAD (0–250 µM) and FMN (0–250 µM) were determined with a fixed concentration of NADH (10 µM). The activity assays for each substrate concentration were done in triplicate, and mean values are given for each substrate concentration. All mean activity values were plotted and adjusted to a Michaelis-Menten model using Excel 2016 (Microsoft Office Professional 2016).

### 2.6. Effects of Temperature and pH on the Activity of Tt-NOX Variants

The activities of soluble Tt-NOX variants were assayed at different temperatures (from 25 to 90 °C) in 50 mM sodium phosphate and at different pH values (pH 5–10) as described in Section 2.5. The following buffer systems (50 mM) were used: sodium acetate (pH 5.0), sodium citrate (pH 6.0), sodium phosphate (pH 7.0 and 8.0) and sodium carbonate (pH 9.0 and 10.0). All pH values were adjusted using a pH-meter with a temperature sensor.

### 2.7. Thermal-stability Assays and Melting-point Determination

Tt-NOX preparations of the different variants (soluble and immobilized) were incubated at different pH values (5, 7, and 9) and temperature values (70 or 80 °C) as indicated in each case. Samples were withdrawn at different time points, and NOX activity was measured as previously described.

The melting point was determined by the ThemoFAD method previously described [28]. Experiments were performed using a MiniOpticon Real-Time PCR detection system (Bio-Rad Laboratories, Hercules, CA, USA) using the fluorescence of the isoalloxazine ring of flavin cofactors. Protein concentration for Thermo FAD analysis was 0.5 mg/mL.

### 2.8. SDS-PAGE Analysis

Sodium dodecyl sulphate polyacrylamide gel electrophoresis (SDS-PAGE) was carried out following the method described previously [26]. Samples were analyzed using 12% polyacrylamide gels under standard denatured conditions.

### 2.9. Quantification of FMN and FAD Bound to the Enzyme

Spectrophotometric determination of the flavin content: Flavin content was determined following the method described previously [29]. 2 mL of hot methanol was added to 0.5 mL of 50 mM sodium phosphate, pH 7, containing 0.25 mg of purified Tt-NOX. The mixture was incubated at 100 °C for 30 min to release non-covalently bound FAD and FMN. Then, it was centrifuged at 12,500 rpm for 15 min at 4 °C. The methanol was evaporated under a stream of N_2_, and the residue was dissolved in 50 mM sodium phosphate, pH 7. Flavin concentration was determined from the absorbance at 450 nm, assuming the ε = 11.3 mM^−1^·cm^−1^.

HPLC identification and quantification of FAD and FMN: FAD and FMN were released from the purified protein as described above. Samples were deproteinized by centrifugation using Amicon Ultra centrifugal tubes with 10 kDa cut-off (Millipore, MA, USA). Flavin content was analyzed by reverse-phase HPLC (Spectra Physic Thermo SP 100 coupled to a UV-vis Spectra System UV 6000 LP diode array detector, Waltham, MA, USA) using a Kromasil C18 column (15 cm × 0.46 cm) supplied by Analisis Vinicos (Tomelloso, Spain). A linear gradient from 100% A (50 mM ammonium acetate, pH 6) to 40% B (acetonitrile) was applied at a flow rate of 0.63 mL/min. The UV detection was performed at 264 nm. The injection volume was set at 20 μL. All experiments were carried out at room temperature. The retention time was 14.4 min for FAD and 15.9 min for FMN.

### 2.10. Computational Details

The X-ray which solved the crystal structure of the Tt8-NOX (PDB id. 1NOX, variant R166/R174/H194) (doi: 10.2210/pdb1nox/pdb) was used as a template to build the other two variants, K166/R174/Y194 and K166/H174/Y194, by chemical modification of the corresponding side chains using the *builder* module in PyMOL 1.8.x. In all cases, the variants were simulated as dimers with a full-oxidized molecule of (2R,3S,4S)-5-(7,8-Dimethyl-2,4-dioxo-3,4-dihydrobenzo[g]pteridin-10(2H)-yl)-2,3,4-trihydroxypentyl dihydrogen phosphate (riboflavin monophosphate, FMN) as a cofactor in each active site. The force field parameters of FMN were obtained at the B3LYP/6-31G* level of theory and the ESP punctual charges were derived by fitting the electrostatic potential. The ground state geometry was optimized in gas phase (B3LYP/3-21G*) with a net charge of –2. Care was taken with the stereocenters in the cofactor. Each complex was embedded in a box of ~16500 TIP3P water molecules [30] that extended 20 Å away from any solute atom and six Cl-ions were added to ensure electrical neutrality. The system was relaxed by energy minimization in three consecutive steps (3 × 5000 cycles) in which after the first 1000 cycles the minimization method was switched from steepest descendent to conjugate graduate. The resulting system was heated from 100 to the target temperature during 20 ps with the position of the solute atoms were restrained (20 kcal·mol^−1^·Å^−2^) using the canonical Langevin thermostat with a Collision frequency of 1 ps^−1^ and with fixed volume (NVT ensemble). These harmonic restraints were gradually reduced in five steps (5 × 20 ps) until they were completely removed. In the latter equilibrating step, the system was changed to an NPT ensemble. The system was further simulated under the same conditions up to a total time of 0.5 µs with a time step of 2 fs without any constraint. System coordinates were printed every 10 ps for further analysis. The cutoff distance for the non-bonded interactions was 10 Å and periodic boundary conditions were used. Electrostatic interactions were treated using the smooth particle mesh Ewald (PME) method. The SHAKE algorithm [31] was applied to all bonds involving hydrogen atoms in the classical region. The molecular dynamics (MD) simulation protocol made use of the pmemd_cuda.SPFP module and the MD simulation trajectories were analysed using the cpptraj module in the AMBER16 suite of programs.(URL; available online: http://ambermd.org/AmberModels.php) All the MD simulations were performed using NVIDIA GPUs.

## 3. Results and Discussion

### 3.1. Expression of the Different Recombinant Tt27-NOX Variants and Sequence Analysis

In a previous work, we reported the isolation, purification, and characterization of the Tt27-NOX and its preliminary optimization for its biotechnological application [10]. The amino acid sequence of the cloned gene presented a 98.5% identity to a similar enzyme isolated from the *T. thermophilus* strain HB8 [3]. After analysis of their amino acid sequences, three divergent residues were found at the positions 166 (R166K), 174 (R174H), and 194 (H194Y) (Figure 1). The biochemical characterization of the Tt27-NOX showed that this variant displayed a higher catalytic efficiency than Tt8-NOX. This difference is probably related to the mutations observed. Moreover, the variant Tt27-NOX (K166/H174/Y194) also differed from the sequence of the strain HB27 published by Henne et al. where a single amino acid changes at position 194 (H194Y) [21]. This difference suggested either an error in the original sequence, or that our strain had adapted to laboratory conditions due to its growth and maintenance. We hypothesized that higher NOX activity might improve bacterial ability to grow in TB medium since this rich medium is likely to promote a reducing power excess (increased NADH/NAD^+^ ratio). The non-detection of the substitution H194Y in samples of *T. thermophilus* strain HB27 from other laboratories would support this assumption.

With the aim of shedding some light on the specific role of these three residues, five variants of the TTC0057 gene of *T. thermophilus* HB27 were constructed (Figure 1):

(i) The R166/R174/H194 variant corresponds to the gene bank accession number CAA42707.1, and it is the variant previously described by Park et al. [3];
(ii) The R166/H174/H194 variant corresponds to the sequence that appears in the BacMap genomic atlas (Available online: http://wishart.biology.ualberta.ca/BacMap/) [32];
(iii) The K166/H174/H194 variant corresponds to the published genome sequence of *T. thermophilus* HB27 by Henne et al. [21] and as it appears in the Kyoto Encyclopedia of Genes and Genomes (KEGG) (Available online: http://www.genome.jp/kegg/kegg2.html);
(iv) The K166/R174/Y194 and (v) R166/H174/Y194 variants correspond to two other mutants that were constructed to identify other possible alterations in the properties of the enzyme.

All the variants were cloned into the pET-22b (+) expression vector and expressed in *E. coli* BL21 DE3. The vast majority of the recombinant NOX variants were obtained in the soluble fraction facilitating its purification.

### 3.2. Thermostability Analysis of Tt27-NOX Variants and its Influence on the Temperature-based Purification Process

The different Tt27-NOX variants cloned in *E. coli* were purified by thermal shock, taking advantage of their thermophilic nature. This purification strategy is a simple way to achieve high purification factors eliminating the vast majority of mesophilic proteins [10,33,34]. To further purify Tt27-NOX variants, two ionic chromatographic steps were applied using PEI-Ag and DS-Ag, which can absorb most of the proteins in an extract of *E. coli* [35,36]. Thus, the three-step purification protocol used was the same as described previously for the KHY variant [10]. Briefly, this procedure consisted of: (i) a thermal shock at 80 °C for 45 min and subsequent centrifugation to remove cell debris; (ii) incubation of the supernatant obtained in Step 1 with Ag-PEI; and finally (iii) incubation of the supernatant obtained in Step 2 with Ag-DS.

In the course of the standard purification procedure for the K166/H174/Y194, K166/H174/H194 (Figure S1a), and R166/H174/H194 variants, the Tt27-NOX activity was mainly found in the supernatant of the cell lysates. On the contrary, during the purification of the R166/H174/Y194 and R166/R174/H194 variants, the Tt27-NOX activity was decreased after the thermal shock, while the variant K166/R174/Y194 was totally inactivated during the heat treatment.

**Figure 2:**
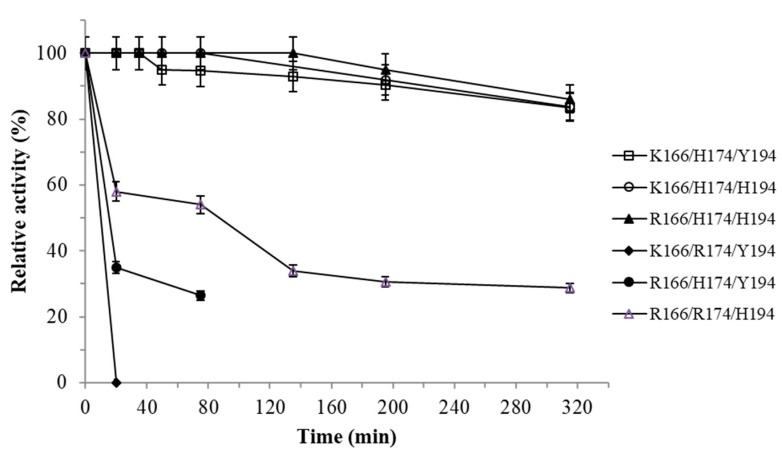
Stability of the soluble Tt27-NOX variants at 80 °C and pH 7. The experiments were carried out in 10 mM sodium phosphate pH 7 under FMN-saturating conditions. Enzymatic activity determined in 10 mM sodium phosphate pH 7, 37 °C, 150 μM FMN, and 0.25 mM NADH.

For this reason, the kinetic and thermodynamic stability of the Tt-NOX variants were determined and compared. As shown in Figure 2, the variant K166/R174/Y194 was completely inactivated after 15 min of incubation at 80 °C. The R166/H174/Y194 variant was the second most unstable variant, retaining 35% of the initial catalytic activity after 20 min of incubation. The third most unstable variant was R166/R174/H194 with a half-life of 75 min. In contrast, the most stable variants were K166/H174/H194, K166/H174/Y194, and R166/H174/H194, retaining 90% of their initial activity after 5 h of incubation.

Therefore, the purification protocol had to be modified to carry out an efficient purification of the K166/R174/Y194, R166/H174/Y194, and R166/R174/H194 variants. In this way, a less severe thermal shock was used for purification of these variants (incubation at 70 °C for 60 min). Under this condition, enzymes retained practically 100% of their initial catalytic activity. Moreover, in the case of the K166/R174/Y194 variant (Figure S1b), the purification procedure omitted the use of the anionic chromatographic support (DS-Ag) to preserve most of the catalytic activity in the supernatant, since at least about 70% of the offered enzyme was able to adsorb onto this support (where the other variants were not significantly adsorbed).

**Figure 3:**
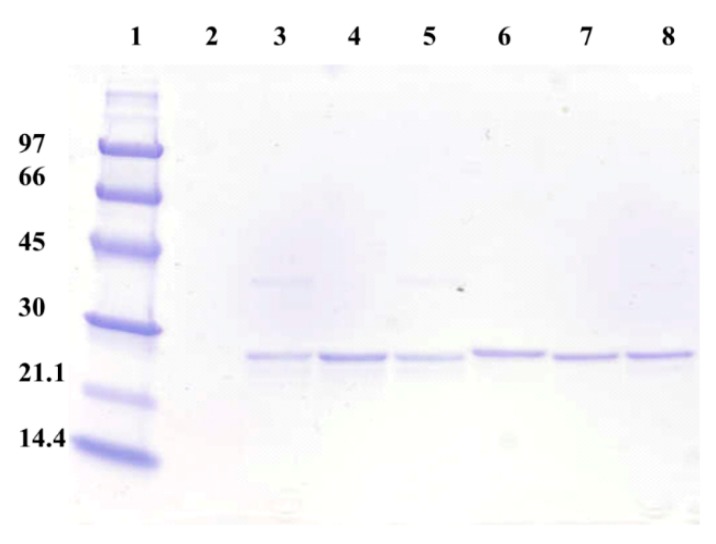
SDS-PAGE analysis of all purified Tt27-NOX variants. Lanes: (1) Molecular weight marker (kDa); (2) Empty lane; (3) K166/H174/Y194 variant; (4) K166/H174/H194 variant; (5) R166/H174/H194 variant; (6) K166/R174/Y194 variant; (7) R166/H174/Y194 variant; and (8) R166/R174/H194 variant.

All the purified Tt-NOX variants (≈ 27 kDa per monomer) are shown in Figure 3. SDS-PAGE analysis showed that the protein band corresponding to the K166/R174/Y194 variant (lane 6) appears slightly above the other variants. It has been found that in certain proteins the electrophoretic mobility depends not only on the molecular weight but also on the net charge, differential binding of the protein to SDS, compactibility of the protein molecules, hydrodynamic form adopted by the protein, etc. [37]. All these results suggest that there is a conformational change in the K166/R174/Y194 variant with regard to the other variants, resulting in a modified electrophoretic mobility (maybe caused by higher interaction with the SDS) and it also allows its adsorption onto this cation exchanger support. This different folding could also lead to some amino acid residues, more sensitive to thermal inactivation, to be more exposed to the solvents.

The changes in enzyme kinetic stability were further confirmed by the determination of melting temperature (*T*_m_) values for the less stable variant (K166/R174/Y194) and one of the most stable and active variants (K166/H174/Y194) (Table 1). The *T_m_* value of K166/H174/Y194 variant was 87.1 °C without the addition of exogenous cofactor, which was 43 °C higher than that of the K166/R174/Y194 variant. In the presence of exogenous flavin cofactor, the *T*_m_ value of the K166/H174/Y194 variant increased approximately 4 °C. In contrast, the addition of flavin cofactor barely changed the *T*_m_ value of the K166/R174/Y194 variant. In the presence of FAD, its *T*_m_ value even decreased slightly. In addition, when thermal stability curves were plotted against normalised fluorescence signal (data not shown), we observed that the K166/R174/Y194 variant could suffer misfolding both in the presence and absence of the exogenous cofactor.

Further differences between these two variants were found when the bound FMN and FAD content was determined (Table S3). Flavin analysis showed that all the mutants contained less of 1 molecule of FMN per subunit and contained practically undetectable levels of FAD. The K166/H174/Y194 variant contained almost 30% of the saturating level of FMN, while the K166/R174/Y194 variant only contained around 5%. Thus, the R174 mutation seemed to reduce the stable binding of FMN by the Tt-NOX active site.

### 3.3. Functional Characterization of Tt27-NOX Variants

**Figure 4:**
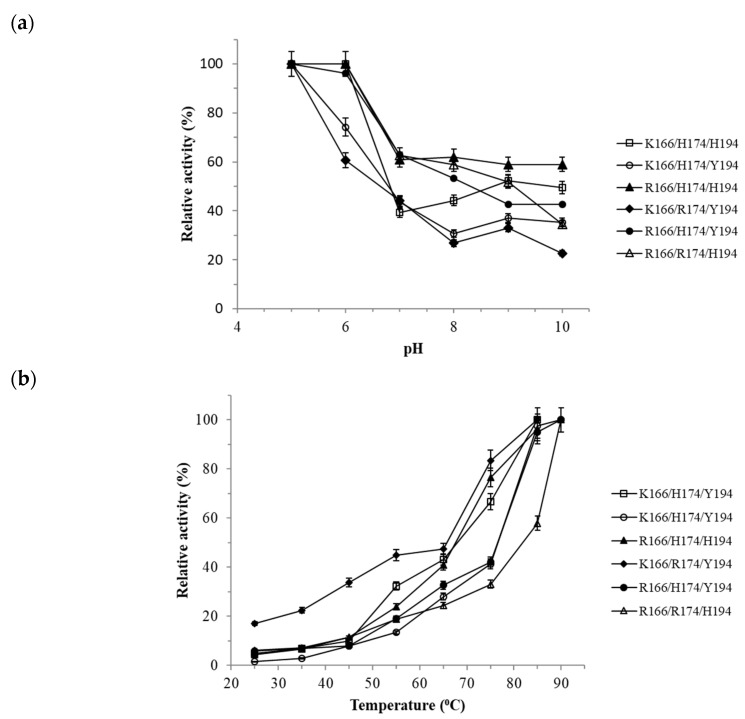
(**a**) The optimal pH of Tt27-NOX variants. The experiments were carried out under FMN-saturating conditions (150 µM), 25 °C, and 0.25 mM NADH. Enzymatic activity determined in pH 5 (sodium acetate 0.1 M); pH 6 (sodium citrate 0.1 M); pH 7 and pH 8 (sodium phosphate 0.1 M); pH 9 and 10 (sodium carbonate 100 mM). Symbols: (□) K166/H174/H194 variant; (○) K166/H174/Y194 variant; (▲) R166/H174/H194 variant; (◆) K166/R174/Y194 variant; (●) R166/H174/Y194 variant; and (△) R166/R174/H194 variant. (**b**) The optimal temperatures of Tt27-NOX variants. The experiments were carried out in 10 mM sodium phosphate pH 7 under FMN-saturating conditions. Enzymatic activity determined in 10 mM sodium phosphate pH 7, 37 °C, 150 μM FMN, and 0.25 mM NADH. Symbols: (●) R166/H174/Y194 variant; (▲) R166/H174/H194 variant; (□) K166/H174/H194 variant; (◆) K166/H174/Y194 variant; (○) K166/R174/Y194 variant; and (△) R166/R174/H194 variant.

The pH profile and temperature dependence of Tt27-NOX variants activity are shown in Figure 4. The optimal enzymatic pH values of Tt-NOX variants were compared as shown in Figure 4a. The optimal pH values of Tt-NOX variants were determined at pH 5. Lower pH values than 5 were not measured due to NADH instability under these conditions [38]. While at alkaline pH, the variants were less active.

The six variants displayed similar activity profiles under different temperatures conditions (Figure 4b). While R166/H174/Y194, R166/H174/H194, KHH194, K166/H174/Y194, and R166/R174/H194 variants showed an optimal temperature of 90 °C, the optimal temperature of the K166/R174/Y194 variant dropped from 90 °C to 80 °C. It was not possible to measure temperatures above 90 °C due to technical problems and the stability of the cofactor. The maximum measured activity was at 90 °C, confirming that it is an extremely active enzyme at high temperatures.

### 3.4. Catalytic Properties Analysis of Tt27-NOX Variants

Tt-NOX is a flavin-dependent oxidase that requires the addition of an exogenous cofactor to reach its maximum activity [10]. The maximum activities, using FAD as a cofactor, were reached between 50 μM (in the case of the R166/R174/H194 variant) and 150 μM FAD (in the case of the K166/H174/H194, K166/H174/Y194, and R166/H174/H194 variants). In contrast, for the K166/R174/Y194 variant, the addition of 300 μM FAD was required to reach the maximum activity. In the case of FMN, the maximum activities were reached at 50 μM for the K166/R174/Y194 variant, 100 μM for the R166/H174/Y194 and R166/R174/H194 variants and 150 μM for variants K166/H174/H194, K166/H174/Y194, and R166/H174/H194.

**Figure 5:**
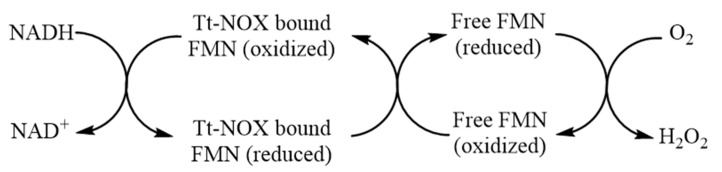
Oxidation of NADH by O_2_ in the presence of Tt-NOX and exogenous free FMN.

The most likely mechanism of NADH oxidation in the presence of free exogenous flavin cofactors is shown in Figure 5, where the presence of flavin cofactor stimulates the oxidase activity of the enzyme. Since free flavin mediates the electron transfer from flavin bound to the enzyme to oxygen [16]:

Steady-state kinetic parameters for Tt27-NOX variants were determined (Table 2). The specific activities in FMN saturating conditions of K166/H174/Y194, K166/H174/H194, R166/H174/H194, R166/H174/Y194, and R166/R174/H194 were 68 ± 3.3, 76 ± 6, 50 ± 2.5, 44.3 ± 2.2, and 42 ± 2.1 U/mg, respectively. In contrast, the K166/R174/Y194 variant showed a specific activity 2.8 and 3.1-fold less than the most active variants, K166/H174/Y194 and K166/H174/H194, respectively.

The affinity of Tt27-NOX variants for NADH was not significantly altered, except for the variant R166/H174/Y194. In the case of R166/H174/Y194, *K*_M NADH_ was increased 3.5-fold compared to K166/H174/H194, which results in lower *k*_cat_/*K*_M_ (6-fold less). Regarding the affinity of the variants for flavin cofactors, it should be noted that K166/R174/Y194 bound 8.5-fold better FMN than FAD. However, the K166/H174/H194 variant showed 4.6-fold higher *k*_cat_/*K*_M_ than K166/R174/Y194.

As shown in Table 3, different NOX microbial proteins have been characterized in some detail. It is important to note that most thermophilic NOXs are producers of hydrogen peroxide. The moderate catalytic activity at low temperatures (25 °C) in the presence of low concentrations of flavin presented by Tt27-NOX variants provides promising biocatalysts for biochemical applications.

### 3.5. Structural Evidences

**Figure 6:**
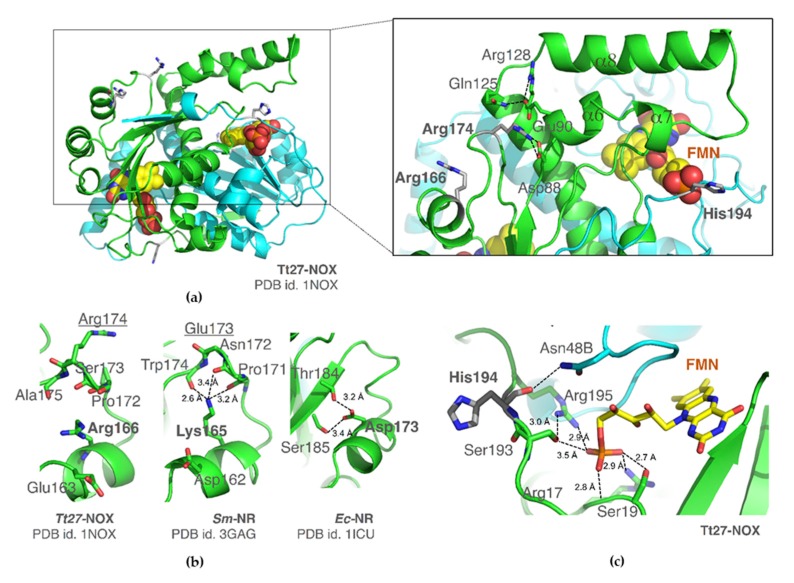
(**a**) NADH oxidase from *Thermus thermophilus*. The two monomers are shown in cartoon in green and in blue, respectively, and the three positions 166/174/194 are colored in grey. On the right hand side, it is shown in detail the mobile domain (α6–α8) and the salt bridge network in which residue 174 (here, an Arg) is implicated. FMN is shown in spheres and colored in yellow. (**b**) Arg166 in Tt27-NOX, equivalent Lys165 in *Sm*-NR Mutants, and equivalent Asp173 in *E. coli* NR. (**c**) Detail of the oxanionic pocket to bind the phosphate group of FMN in Tt27-NOX. Residue His194 is in close proximity and its carbonyl oxygen is hydrogen bonded with the amide of the conserved Asn48B of the other subunit.

To date, only a crystal structure of this enzyme is available in the Protein Data Bank (variant RRH, PDB id. 1NOX) that is in complex with flavin mononucleotide (FMN) as a cofactor [4]. In Figure 6, the dimer is shown in cartoon, and residues 166/174/194 are highlighted. Arg166 is localized on helix α10; Arg174 and His194 are found in α10-β4 and β4-α11 loops, respectively. The former two residues are part of a β-hairpin that includes the short α-helix α10 (Figure S2). Although none of these residues interacts directly with FMN, they all surround the lid that covers the active site (motif α6-α7-α8).

#### 3.5.1. Structural Superimposition. Residue 166

This NADH oxidase from an extreme thermophile shows high sequence similarity with nitroreductases. Indeed, in a structural alignment of Tt27-NOX using the Dali Server (URL; available online: http://ekhidna2.biocenter.helsinki.fi/dali) the best 50 results correspond to 10 nitroreductases (NR) from different species (Table S4). In these enzymes, the nature of the residue in the equivalent position 166 varies, although in all cases this amino acid is enrolled in a hydrogen bond network to fix the β-loop of the β-hairpin where the residue 174 is located. Tt27-NOX is the only enzyme that has an arginine in this location (Figure 6b). The Arg166 side chain is oriented to Glu163. The putative NAD(P)H nitroreductase from *Bacillus subtilis* (*Bs*-NR, PDB id. 3BEM), as well as the nitroreductase-like protein smu.346 from *Streptococcus mutans* (*Sm*-NR, PDB id. 3GAG, doi: 10.2210/pdb3gag/pdb), have a positively-charged lysine residue (Lys165). In the latter case, the side chain of Lys165 is hydrogen bonded with up to three backbone oxygen atoms to stabilize the Pro171-Trp174 β-loop (*Sm*-NR, Figure 6b). In Tt27-NOX, the Arg174 is located on this motif (Glu173 in *Sm*-NR).

It is noteworthy that in chain A of the structure of *Bs*-NR, Lys175 has been assigned with two conformers, and in one of them establishes a salt-bridge with carboxylate of Asp162, with an orientation that is similar to Arg166 in Tt27-NOX. Therefore, a stable interaction between Arg166 and Glu163 in variant R166/H174/Y194 may affect the stability of the Pro172-Ala175 β-loop, and thereby, the thermal stability of R166/H174/Y194. On the contrary, in variant K166/H174/Y194, Lys166 may stabilize the loop in which His174 is located, thus increasing the thermal stability.

Finally, in the nitroreductases from *Eneterobacter cloacae* (*Ec*-NR) [45], *Escherichia coli* BL21 (*Ec*-NR) [46], or *Idiomarina loihiensis* L2TR (*Il*-NR, doi: 10.2210/pdb3of4/pdb), this Arg/Lys is replaced by an aspartic residue (Asp173, Figure 6b), or by Asn162 in the isoform of *Streptococcus pneumoniae TIGR4* (*Sp*-NR, doi: 10.2210/pdb2b67/pdb).

#### 3.5.2. Residue 174

The Arg174 residue forms a salt bridge with the side chain of the highly conserved Asp88 in Tt27-NOX (Figure 6a). The side chain of Glu90 (that is located on helix α6) is found in close proximity. Glu90 establishes a salt-bridge with the Arg128 through its side chain, and a hydrogen bond with the amide of Gln125. All together, these interactions may control the mobility of the motif α6-α7-α8 as an elbow in an arm. In the structural-related nitroreductases of Table S4, there is again high variability. Interestingly, four of them have an Arg in this position and *Sp*-NR (PDB id. 2HAY) have a Lys (Figure S3). In this case, the effect that the introduction of a His residue in this position has is not obvious.

#### 3.5.3. Residue 194

Finally, His194 is placed between Ser193 and Arg195, and its side chains define the oxyanion hole for the recognition of the phosphate group of the cofactor (Figure 6c). A net of interactions with residues Ser19, Arg17, Ser193, and Arg195 recognizes the FMN phosphate oxygens. On the other hand, the backbone oxygen of His194 is hydrogen bonded with the side chain of the conserved residue Asn48. In the other NRases, this His194 is mostly an aliphatic residue (e.g., Val), although *Bs*-NR (PDB id. 3BEM, doi: 10.2210/pdb3bem/pdb) and *Sp*-NR have a Tyr in this position (Tyr190 and Tyr195, respectively). Since the side chain of residue 194 is projected outwards, the substitution of His by Tyr is also not obvious.

#### 3.5.4. Unfolding of Tt27-NOX by Means of Unbiased Extensive Molecular Dynamic Simulations

Since the punctual change of the side chain of residue 174 (His → Arg) has an enormous effect on the melting temperature of the Tt27-NOX variant (91.8 → 43.5 °C), we were committed to investigate the differential thermal unfolding of these two variants by means of extensive unbiased MD simulations. In the crystal structure of NADH oxidase from *Thermus thermophilus* in complex with FMN (RRH, PDB id. 1NOX), the Arg174 side-chain participates in an extended salt bridge network (Figure S2). Located in the loop of the β-hairpin β3- β4, its guanidinium group is bonded to the carboxylate of Asp88 that is part of the polar helix α6 with two other two acid groups upstream: Glu90, which is bonded with Arg128 (α8), and Asp91 binds to Arg53 (β1 strand) and to Arg200′ (C-terminus) of the other subunit. All together, these interactions have been thought to stabilize the conformation of helix α6 [9,47]. However, only the salt bridge between Glu90 and Arg128 has been found to have large temperature-dependent (up to 50 °C) changes in the nitrogen backbone ^15^N chemical shift [48].

**Figure 7:**
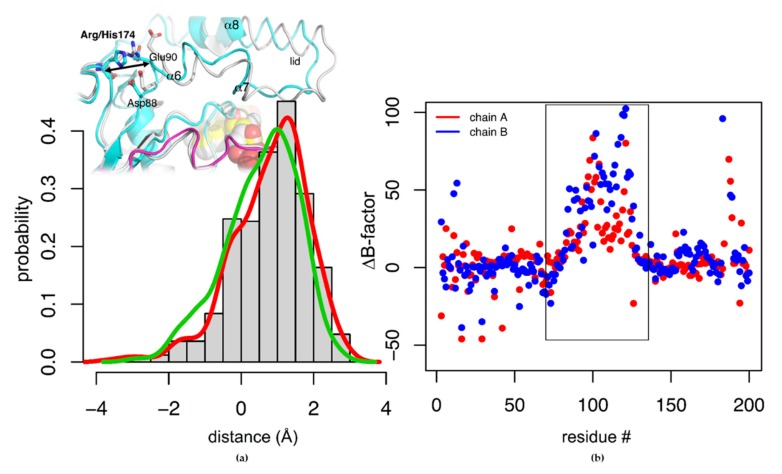
(**a**) Histogram of the difference between distances of alpha carbons of residues Arg/His174 and Glu90 in variant K166/R174/Y194 with respect to K166/H174/Y194 along the MD simulations at 363 K. Both active sites A (red line) and B (green line) are shown. (**b**) B-factor differences per residue along with the MD simulations at 363 K, defined as B-factorKRY-B-factorKHY. A more positive value indicates a more mobile residue along with the MD simulation.

The two variants K166/R174/Y194 and K166/H174/Y194 in complex with FMN as a cofactor (care was taken of its stereochemistry, see Materials and Methods) were simulated for 0.5 µs at different temperatures without introducing any restraint. At the experimental *T*_m_ of the thermostable variant K166/H174/Y194 (90 °C, 363 K), no unfolding was detected even for the thermolabile counterpart (Figure S4). Thus, it was required to reach more than 200 °C (500 K) to see a net loss of secondary structure. Along with the MD simulation at 500 K, there is a significant conformational change in the protein, especially in the residues that define the lid that covers the active site (Figure S4). The percentage of global native contacts drops down up to 15% (Figure S5). Although both variants unfold at this temperature, it is observed that the thermolabile variant presents a bigger conformational change than the themoresistant one (Figure S5). Looking at position 174, both His and Arg side chains interact with Asp88 in a similar fashion. The longer side chain in Arg shifts the distance between residue 166 and helix α6 in ~1–2 Å in KRY variant, as shown by the MD simulation (Figure 7a). Noteworthy, this fact increases the mobility of the lid over the active site (α6-loop-α7-loop-α8, residues 88–123) as it is shown in the differences of B-factor between both variants (Figure 7b). On the other hand, the salt bridge between Glu90 and Arg128 is lost in almost half of the simulation time in both cases, whereas the interaction of Arg128 with the Asp88 carboxylate is kept in both cases, as reported previously in other shorted MD simulations of the R166/R174/H194 variant [9,47].

#### 3.5.5. Cofactor Specificity

**Figure 8:**
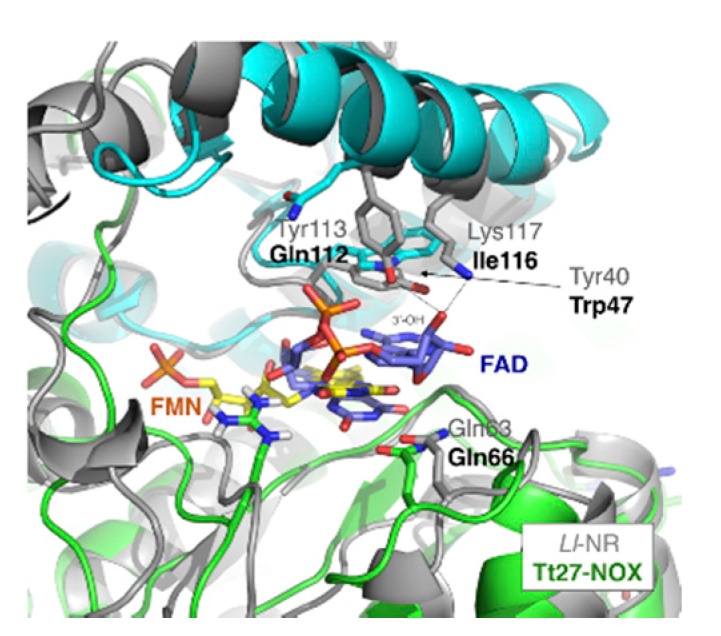
Structural superimposition of nitroreductase from Idiomarina loihiensis L2TR (Ll-NR) and Tt27-NOX.

The chemical differences between the two cofactors flavin mononucleotide (FMN) and flavin adenine dinucleotide (FAD) relays on the presence of an extra 5′-phosphate adenine moiety bound to the phosphate group that is present in FMN. Figure 8 shows the structural superimposition of Tt27-NOX with its structural-analog NR from *Idiomarina loihiensis* L2TR co-crystallized with FAD. In both cases, the recognition of the flavin moiety is the same. In the case of FAD, the ribose is kept by hydrogen bonds of 3′-OH to both Tyr113 and Lys117 side chains. The two equivalent residues in Tt27-NOX are Gln112 and Ile116 that are located on helix α8. Tyr40 (Trp47 in Tt27-NOX) interacts with one of the oxygen O1P. Again, the reason why K166/R174/Y194 shows 8.5 times more affinity for FMN than for FAD is due to an increase in the mobility of the lid, which may affect the former recognition pattern of the adenosine moiety. In contrast, we observe in our simulations that the interactions at the oxanionic hole with the phosphate group of FMN are preserved even in the unfolded conformations of the K166/R174/Y194 variant at 500 K.

## 4. Conclusions

In this study, five variants of the Tt27-NOX (K166/H174/H194, K166/H174/Y194, K166/R174/Y194, and R166/H174/H194) were characterized and compared to the Tt8-NOX variant (R166/R174/H194) reported in the literature [3]. Two highly thermostable and active Tt27-NOX mutants were obtained by the substitutions R166K and R174H. The effect of the substitution of His to Tyr residue at the position 194 was not obvious. The variants K166/H174/Y194 and K166/H174/Y194 retained 90% of their initial activity after 5 h at pH 7 and 80 °C. In contrast, the variant K166/R174/Y194 was inactivated after 15 min of incubation under the same conditions. The maximum measured activity was at 90 °C with the exception of the K166/R174/Y194 variant, which presented an optimum temperature of 80 °C. The optimal pH value was 5 for all the variants.

The thermoresistant variants were also the most catalytically active. Thus, the catalytic efficiency of the variants K166/H174/H194 and K166/H174/Y194 was 4.6 and 3.2-fold higher than the K166/R174/Y194, respectively. Remarkably, the introduction of an Arg residue at position 174 produced an 8.5-fold more affinity for FMN than FAD. The presence of Arg174 seems to facilitate a higher mobility of the lid over the active site (α6-loop-α7-loop-α8, residues 88–123), which could be responsible for the modification of the cofactor specificity.

Therefore, these results indicated that the catalytic properties of the Tt-NOX could be further improved by protein engineering. Its high thermostability and moderated activity at low temperatures (25 °C) in the presence of low flavin concentrations provide a promising biocatalyst that has already been successfully applied in cofactor regeneration, in situ enzymatic production of hydrogen peroxide, activation of chemotherapeutic agents, and as a biosensor.

## Figures and Tables

**Table 1:** Thermodynamic stability (*T*_m_) of the K166/R174/Y194 and K166/H174/Y194 variants in the presence and the absence of flavin cofactors.

Tt-NOX Variants	*T*_m No flavin Cofactor_ (°C)	*T*_m FMN_ (°C)	*T*_m FAD_ (°C)
K166/R174//Y194	44.1	43.5	39.8
K166/H174/Y194	87.1	91.8	90.8

The melting temperature was determined as described in the Methods section.

**Table 2:** Apparent kinetic parameters of the purified Tt27-NOX variants calculated at pH 7 and 25 °C.

Tt-NOX Variants	*K*_M NADH_ (µM) ^1^	*K*_M FMN_ (µM) ^2^	*K*_m FAD_ (µM) ^2^	*K*_M Riboflavin_ (µM) ^2^	Specific Activity^c^ (U/mg) ^3^	*k*_cat NADH_ (s^−1^)	*k*_cat NADH_/*K*_M NADH_ (10^6^ M^−1^·s^−1^)
K166/H174/Y194	1.6 ± 0.1	43 ± 2.2	34 ± 1.7	69.7 ± 3.5	68 ± 3.3	23.3 ± 1.2	14.6 ± 0.07
K166/H174/H194	1.3 ± 0.06	45 ± 2.3	46 ± 2.3	68.7 ± 3.4	75 ± 6	27.2 ± 1.4	21 ± 1.1
R166/H174/H194	1.3 ± 0.06	47 ± 2.3	29 ± 1.5	50.7 ± 2.6	50 ± 2.5	17.9 ± 0.9	13.8 ± 0.7
K166/R174/Y194	1.9 ± 0.1	15 ± 0.8	127 ± 6.4	60 ± 3	24 ± 1.2	8.6 ± 0.4	4.5 ± 0.22
R166/H174/Y194	4.6 ± 0.2	27 ± 1.4	30 ± 1.5	44 ± 2.2	44.3 ± 2.2	15.9 ± 0.8	3.5 ± 0.18
R166/R174/H194	1.8 ± 0.1	23.6 ± 1	31.9 ± 1.4	50.8 ± 2.6	42 ± 2.3	15.1 ± 0.8	8.4 ± 0.42

The steady-state kinetics parameters were calculated at pH 7 and 25 °C. Activities were adjusted to a non-linear regression. ^1^ Kinetic parameters for NADH were calculated using 50 µM FAD. ^2^ Kinetic parameters for flavin cofactors were calculated using 10 µM NADH. ^3^ Specific activities were determined at 25 °C, pH 7, and 200 µM FMN.

**Table 3:** Summary of published data from characterization of NOXs enzymes from different organisms.

NOX Origin	NOX Type 1=H_2_O_2_; 2=H_2_O	Specific Activity _NADH_ (U/mg)	*K*_M NADH_ (µM)	pH Optimum	Temperature Optimum (°C)	Reference
*T. thermophilus* HB27 (K166/H174/H194)	2	75 (25 °C)	1.3	5	90	This work
*T. thermophilus* HB8	2	5.2 (25 °C)	4.14	5	80	[3]
*Thermotoga. maritima*	2	230 (80 °C)	42	n.d.	80	[39]
*Clostridium thermosaccharolyticum*	2	23 (25 °C)	19	7	n.d.	[40]
*Archaeogiobus fulgidus* (NoxA-1)	2	5.82 (70 °C)	0.13	8	80	[41]
*Thermococcus kodakarensis* KOD1	2	0.38 (25 °C)	49	3.5	> 90	[42]
*Thermococcus profundus*	1, 2	7 (75 °C)	53.1	7.5–8.0	70	[43]
*Pyrococcus furiosus*	1, 2	20 (75 °C)	< 4	5.5–8.5	85	[44]

n.d. Not described.

## Supplementary Materials

The following are available online at https://www.mdpi.com/2076-2607/7/11/515/s1, Figure S1: SDS-PAGE analysis of the purification process of the a) K166/H174/H194 and b) K166/R174/Y194 variants. a) Lanes: 1) Molecular weight marker (kDa); 2) Crude protein extract; 3) Supernatant after heat treatment at 80 °C for 45 minutes; 4) Supernatant after incubation in presence of PEI-Ag for 45 minutes; 5) Supernatant after incubation in the presence of DS-Ag for 45 minutes. b) Lanes: 1) Molecular weight marker (kDa); 2) Empty lane; 3) Crude protein extract; 4) Supernatant after heat treatment at 70 °C for 60 minutes; 5) Supernatant after incubation in presence of PEI-Ag for 45 minutes., Figure S2: Sequence alignment of enzymes included in Table S1, Figure S3: Hydrogen bond network of Arg174 and Arg166 of Tt27-NOX (PDB id. 1NOX). The two monomers of the enzyme are colored in green and in blue, respectively. FMN is shown in sticks, Figure S4: RMSd (Å) evolution of the two variants K166/H174/Y194 and K166/R174/Y194 along 0.5 µs of unbiased MD simulation, Figure S5: Native contacts (%) evolution of the two variants K166/H174/Y194 and K166/R174/Y194 along 0.5 µs of unbiased MD simulations at different temperatures. Together with the global number of native contacts, the evolution of the residues of the lid is also shown; Table S1: Primers used in this study, Table S2: Plamids used in this study, Table S3: Quantification of flavin cofactor content, Table S4: Structural alignment of Tt27-NOX using Dali Server. Best 50 results.
