# Supplementary material for: Functional Characterization and Structural Analysis of NADH Oxidase Mutants from Thermus thermophilus HB27: Role of Residues 166, 174, and 194 in the Catalytic Properties and Thermostability
Source: Microorganisms. 2019 Oct 31;7(11):515. doi: 10.3390/microorganisms7110515 (PMC6921046; doi:10.3390/microorganisms7110515)
Supplement: Supplementary file 1 [file microorganisms-07-00515-s001.pdf]

Supplementary information

Table S1. Primers used in this study.

Name	Sequence (5´ ⟶ 3´)
**KHY-F**	ttc<u>catatg</u>atggaggcgacccttcc
**KHY-R**	ttc<u>gaattc</u>ctagcgccagaggaccac
**Y194H-F**	gctacccctcccaccgcctgccc
**Y194H-R**	gggcaggcggtgggaggggtagc
**K166R-F**	tgaccccgagagggtgagggcgatcct
**K166R-R**	aggatcgccctcaccctctcggggtca
**H174R-F**	ggcttccttcccgcgccgccatccc
**H174R-R**	gggatggcggcgcgggaaggaagcc

Restrictions sites are shown underlined.

Table S2. Plamids used in this study.

Plasmid	Description	Reference
**pET22b(+)**	Amp^r, lacl. Expression of genes in *E. coli*, dependent of T7 phague RNA polymerase	Novagen
**pET22b-KHY**	Amp^r. NOX overexpression without C-terminal His-tag (NdeI/EcoRI). DNA fragment (TTC0057 gene from *T. thermophilus* HB27) amplified with KHY-F and KHY-R primers	[1]
**pET22b-KHH**	Amp^r. KHH variant overexpression without C-terminal His-tag (NdeI/EcoRI). The mutation was introduced with Y194H-F and Y194H-R primers, replacing Y/H in Nox postion 194 and using pET22b-KHY as a template	This study
**pET22b-RHY**	Amp^r. RHY variant overexpression without C-terminal His-tag (NdeI/EcoRI). The mutation was introduced with K166R-F and K166R-R primers, replacing K/R in NOX position 166 and using pET22b as a template	This study
**pET22b-KRY**	Amp^r. KRY variant overexpression without C-terminal His-tag (NdeI/EcoRI). The mutation was introduced with H174R-F and H174R-R primers, replacing H/R in Nox position 174 and using pET22b-KHY as a template	This study
**pET22b-RHH**	Amp^r. RHH variant overexpression without C-terminal His-tag (NdeI/EcoRI). The mutation was introduced with K166R-F and K166R-R primers, replacing K/R in NOX position 166 and Y/H in NOX position 194 and using pET22b-KHH as a template	This study
**pET22b-KRH**	Amp^r. RRH variant overexpression without C-terminal His-tag (NdeI/EcoRI). The mutation was introduced with H174R-F and H174R-R primers and using pET22b-KHY as a template.	This study
**pET22b-RRH**	Amp^r. RRH variant overexpression without C-terminal His-tag (NdeI/EcoRI). The mutation was introduced with K166R-F and K166R-R primers and using pET22b-KRH as a template.	This study

Table S3. Quantification of flavin cofactor content.

Tt-NOX variant	Cofactor	HPLC method (μM)	Spectrophotometric method (μM)	Enzyme molecules containg flavin cofactor (%)
K166/R174/Y194	FAD	traces	ND	-
	FMN	1.15	1.1	5.5
K166/H174/Y194	FAD	traces	ND	-
	FMN	5.6	5.84	29

Experiments were performed with 20 μM of purified protein. Flavin content was determined by HPLC and spectrophotometric method as described in methods section. ND: non-detectable.

Table S4. Structural alignment of Tt27-NOX using Dali Server. Best 50 results.

PDB id.	UNIPROT id.	Chain	Resolution (Å)	Name	Organism	Z-value	RMSd (Å)	id (%)
1nox	Q60049	A	1.59	NADH oxidase		33.6	0.0	100
3gbh	A0A0H2VHN8	A	2.00	Putative NAD(P)H:FMN Oxidoreductase	*Staphylococcus epidermidis ATCC 12228*	23.9	1.8	23
3bem	P96692	A	1.65	Putative NAD(P)H Nitroreductase YDFN	*Bacillus subtilis*	23.5	1.7	24
3ge6	B1YG32	A	1.70	Nitroreductase	*Exiguobacterium sibiricum*	23.3	2.0	28
3of4	Q5R179	B	1.90	FMN/FAD- and NAD(P)H-dependent Nitroreductase	*Idiomarina loihiensis L2TR*	23.2	1.9	21
3gag	Q8DVW4	B	1.70	Nitroreductase-like protein (smu.346)	*Streptococcus mutans*	22.6	2.1	25
2b67	A0A0H2UP38	B	1.90	Nitroreductase	*Streptococcus pneumoniae TIGR4*	22.3	2.3	27
4qly	U6C5W9	D		Enone reductase CLA-ER	*Lactobacillus plantarum*	22.1	2.4	23
**1nec**<br>1kqb<br>1kqc<br>1kqd<br>5j8d<br>5j8g	Q01234	A	1.95	Nitroreductase	*Eneterobacter cloacae*	20.2	2.6	25
**1icu**<br>3x22<br>3x21<br>1yki<br>1ylr<br>1ylu<br>1idt<br>1oo5<br>1oo6<br>1oon<br>1ooq<br>1icr<br>1icv<br>1ds7<br>1idt	P38489	C	1.80	Nitroreductase	*Escherichia coli BL21*	20.2	2.6	24
2hay	Q9A120	B	2.11	Putative NAD(P)H-Flavin Oxidoreductase	*Streptococcus pyogenes*	20.0	2.3	24

a)

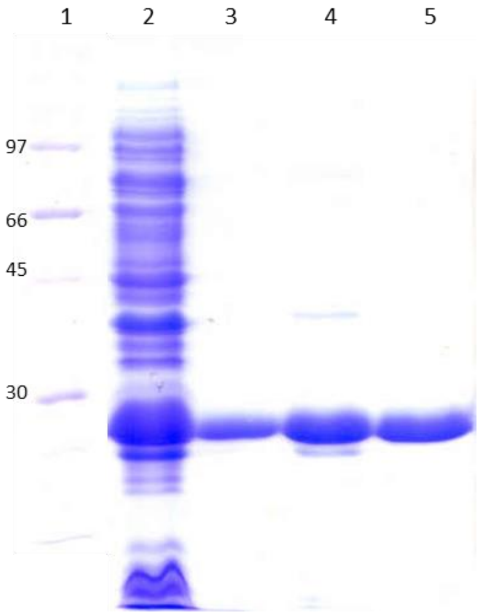

b)

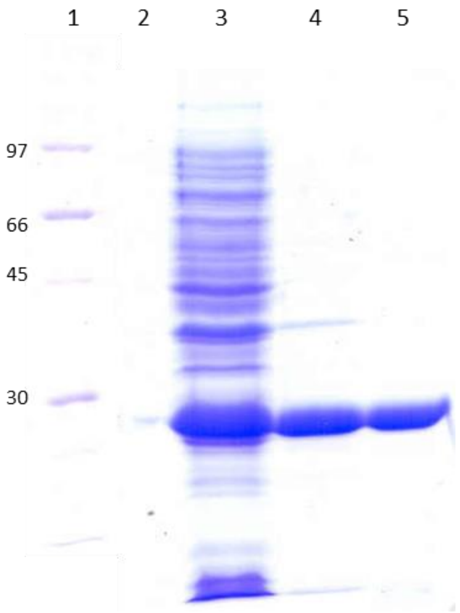

Figure S1. SDS-PAGE analysis of the purification process of the **a)** K166/H174/H194 and **b)** K166/R174/Y194 variants. **a)** Lanes: 1) Molecular weight marker (kDa); 2) Crude protein extract; 3) Supernatant after heat treatment at 80 °C for 45 minutes; 4) Supernatant after incubation in presence of PEI-Ag for 45 minutes; 5) Supernatant after incubation in the presence of DS-Ag for 45 minutes. **b)** Lanes: 1) Molecular weight marker (kDa); 2) Empty lane; 3) Crude protein extract; 4) Supernatant after heat treatment at 70 °C for 60 minutes; 5) Supernatant after incubation in presence of PEI-Ag for 45 minutes.

```
sp|Q60049|NOX_THET8                 ---MEATLPVLDAKTAA-LKRRSIRRYRKD-PVPEGLLREILEAALRAPSAWNLQPWRIV     55
tr|A0A0H2VHN8|A0A0H2VHN8_STAES      ---MQKLTRINDFNEVL-NSRKSVKVFDENYKIPREEMDEIITKATKAPSSVNMQPWRIA     56
sp|P96692|MHQN_BACSU                ---------MAEFTHLV-NERRSASNFLSGHPITKEDLNEMFELVALAPSAFNLQHTKYV     50
tr|B1YG32|B1YG32_EXIS2              ----MTTQTATDFMEIV-KGRRSIRNYDTNVKISKEEMTQILEEATLAPSSVNMQPWRFL     55
tr|Q5R179|Q5R179_IDILO              -----------MYLEKLQQWRYATADF-SGAHITDDVLDKLLNTTRLTASSYGLQPYCTL     48
tr|Q8DVW4|Q8DVW4_STRMU              --------MMNDYLNFL-DGRVSVRQFDPDAVLPNDLIKDMLEHASYAPSGNNFQPWRVV     51
tr|A0A0H2UP38|A0A0H2UP38_STRPN      -------MKFL---ELN-KKRHATKHFTDK-LVDPKDVRTAIEIATLAPSAHNSQPWKFV     48
tr|U6C5W9|U6C5W9_LACPN              MSEAVKNLVNNDLADVM-FNRHSVRQFDPNVKIGRDELQKMIAEAATAPSACNLQSWHFV     59
sp|Q01234|NFSB_ENTCL                ----------MDIISVA-LKRHSTKAFDASKKLTAEEAEKIKTLLQYSPSSTNSQPWHFI     49
sp|P38489|NFSB_ECOLI                ----------MDIISVA-LKRHSTKAFDASKKLTPEQAEQIKTLLQYSPSSTNSQPWHFI     49
tr|Q9A120|Q9A120_STRP1              ----MDQTIHHQIQQAL-HFRTAVRVYKEE-KISDEDLALILDAAWLSPSSIGLEGWRFV     54
                                                        * :   :     :              : *. . :

sp|Q60049|NOX_THET8                 VVRDPATKRALRE-----AAF-GQAHVEEAPVVLVLYADLEDALAHLDEVI-HPGVQGER     108
tr|A0A0H2VHN8|A0A0H2VHN8_STAES      VVQSDEMKEKVKE----SFGF-NSRQLTTSSAMLIIFGDLQNYEKAEQIYG-DAVEQQLM     110
sp|P96692|MHQN_BACSU                TVLDQDVKEKLKQ-----AAN-GQYKVVSSSAVLLVLGDKQAYQQAADIYE-GLKVLGIL     103
tr|B1YG32|B1YG32_EXIS2              VIDSEEGKATLAP----LAKF-NQVQVETSSAVIAVFGDMKAIDQLENIYD-TAVEKGLM     109
tr|Q5R179|Q5R179_IDILO              VIRNKGLREQLVN-----HSF-GQQKVADSSALVIFAAKTGAVADIVDPYISELSQQRQL     102
tr|Q8DVW4|Q8DVW4_STRMU              VVKNKNKQEDLKK-----LAA-LQPQVATASAVFLLFGDENAYDLT-WWQE-FHVQKGII     103
tr|A0A0H2UP38|A0A0H2UP38_STRPN      VVREKNAELAKLA-----YGS-NFEQVSSAPVTIALFTDTDLAKRAR-KIA-RVGGANNF     100
tr|U6C5W9|U6C5W9_LACPN              VVDTPEAKAKFKQA---VMKF-NYPQVDSASAIVFIAGDTQSHYVYRDVWN-KVYEDGNI     114
sp|Q01234|NFSB_ENTCL                VASTEEGKARVAKSAAGTYVF-NERKMLDASHVVVFCAKTAMDDAWLERVVDQEEADGRF     108
sp|P38489|NFSB_ECOLI                VASTEEGKARVAKSAAGNYVF-NERKMLDASHVVVFCAKTAMDDVWLKLVVDQEDADGRF     108
tr|Q9A120|Q9A120_STRP1              VLDNKPIKEEIKP-----FAWGAQYQLETASHFILLIAEKHARYDSPAIKN-SLLRRGIK     108
                                    .      .                 ::  :   . .  .

sp|Q60049|NOX_THET8                 REAQKQ----AIQRA-----FAAMG---QEARKAWASGQSYILLGYLLLLLEAYGLGSVP     156
tr|A0A0H2VHN8|A0A0H2VHN8_STAES      TEDIKAQLLDWILPY-----YKNLS---REGMKDIVNIDSSLMAMQLMLTAKAHGYDTNP     162
sp|P96692|MHQN_BACSU                NKQEYDHMVQDTVSF-----YENRG---EQFKRDEAIRNASLSAMMFMLSAKEKGWDTCP     155
tr|B1YG32|B1YG32_EXIS2              PQEVRDRQVPAIQGM-----YENVP---ASALKDSILIDSGLVSMQLMLVARAHGYDTNP     161
tr|Q5R179|Q5R179_IDILO              TNEEAENTRNYFTQK-----LQAMS---AATRKEWAVRQAYIGLGTFLLAAAELEVDSCP     154
tr|Q8DVW4|Q8DVW4_STRMU              TKDEAAARAERIRQY-----FDLHP---EDKETQGLRLDVGLFAMNLMQVVRVYGYDSVP     155
tr|A0A0H2UP38|A0A0H2UP38_STRPN      SEEQLQYFMKNLPAE-----FARYS---EQQVSDYLALNAGLVAMNLVLALTDQGIGSNI     152
tr|U6C5W9|U6C5W9_LACPN              TKERLDQILGTFLPL-----YENAT---PDFLKFDATIDCSVVGMQLLLVARAHGYDANA     166
sp|Q01234|NFSB_ENTCL                NTPEAKAANHKGRTY-----FADMHRVDLKDDDQWMAKQVYLNVGNFLLGVGAMGLDAVP     163
sp|P38489|NFSB_ECOLI                ATPEAKAANDKGRKF-----FADMHRKDLHDDAEWMAKQVYLNVGNFLLGVAALGLDAVP     163
tr|Q9A120|Q9A120_STRP1              EGDGLNSRLKLYESFQKEDMDMADN---PRALFDWTAKQTYIALGNMMMTAALLGIDTCP     165
                                                                          :  :    ::        .:

sp|Q60049|NOX_THET8                 MLGFDPERVRAILGL-----PSHAAIPALVALGYPAEEGY----PSHRLPLERVVLWR- 205
tr|A0A0H2VHN8|A0A0H2VHN8_STAES      IGGFDKENIADIIGYD----SDRYVPVLAIAIGKKAQDAH----DSVRLPIDDVREFL- 212
sp|P96692|MHQN_BACSU                MIGFDAEAVKRILNI-----DDQFEVVMMITIGKEKTESRR--PRGYRKPVNEFVEYM- 206
tr|B1YG32|B1YG32_EXIS2              IGGYEKDQIAEAFGME----KDRYVPVMLLSIGKAVDAGY----PSVRLPINDIADWK- 211
tr|Q5R179|Q5R179_IDILO              MEGIEHDAYDNILSLK----DLGLSTVFACPVGYRSEADTTQFQKKVRQPLSRFKVVL- 208
tr|Q8DVW4|Q8DVW4_STRMU              MRGVDFDAIKTYLDM-----PNEWEPILMLPVGKALQAGN----PHVRKSVAEFAEIIE 205
tr|A0A0H2UP38|A0A0H2UP38_STRPN      ILGFDKSKVNEVLEI-----EDRFRPELLITVGYTDEKLE----PSYRLPVDEIIEKR- 201
tr|U6C5W9|U6C5W9_LACPN              FSGIDFEKMIPTLGLD----PKRYVPVMGIAIGKAAQEPL----HTTRYDAKTQTDFLA 217
sp|Q01234|NFSB_ENTCL                IEGFDAAILDEEFGLK----EKGFTSLVVVPVGHHSVEDFNATLPKSRLPLSTIVTEC- 217
sp|P38489|NFSB_ECOLI                IEGFDAAILDAEFGLK----EKGYTSLVVVPVGHHSVEDFNATLPKSRLPQNITLTEV- 217
tr|Q9A120|Q9A120_STRP1              IEGFHYDKVNHILAKHNVIDLEKEGIASMLSLGYRLRDPKH---AQVRKPKEEVISVVK 221
                                    : * .       :                  :*              *
```

Figure S2. Sequence alignment of enzymes included in Table S1.

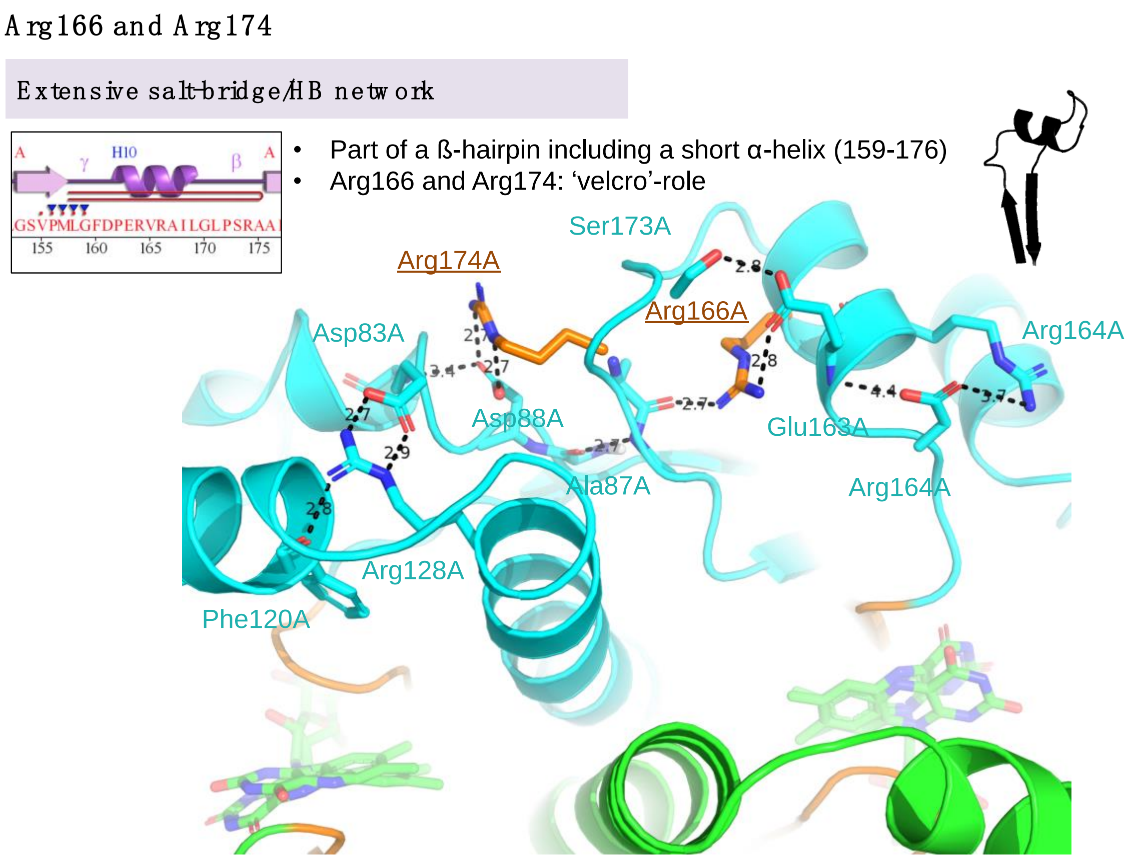

Figure S3. Hydrogen bond network of Arg174 and Arg166 of Tt27-NOX (PDB id. 1NOX). The two monomers of the enzyme are colored in green and in blue, respectively. FMN is shown in sticks.

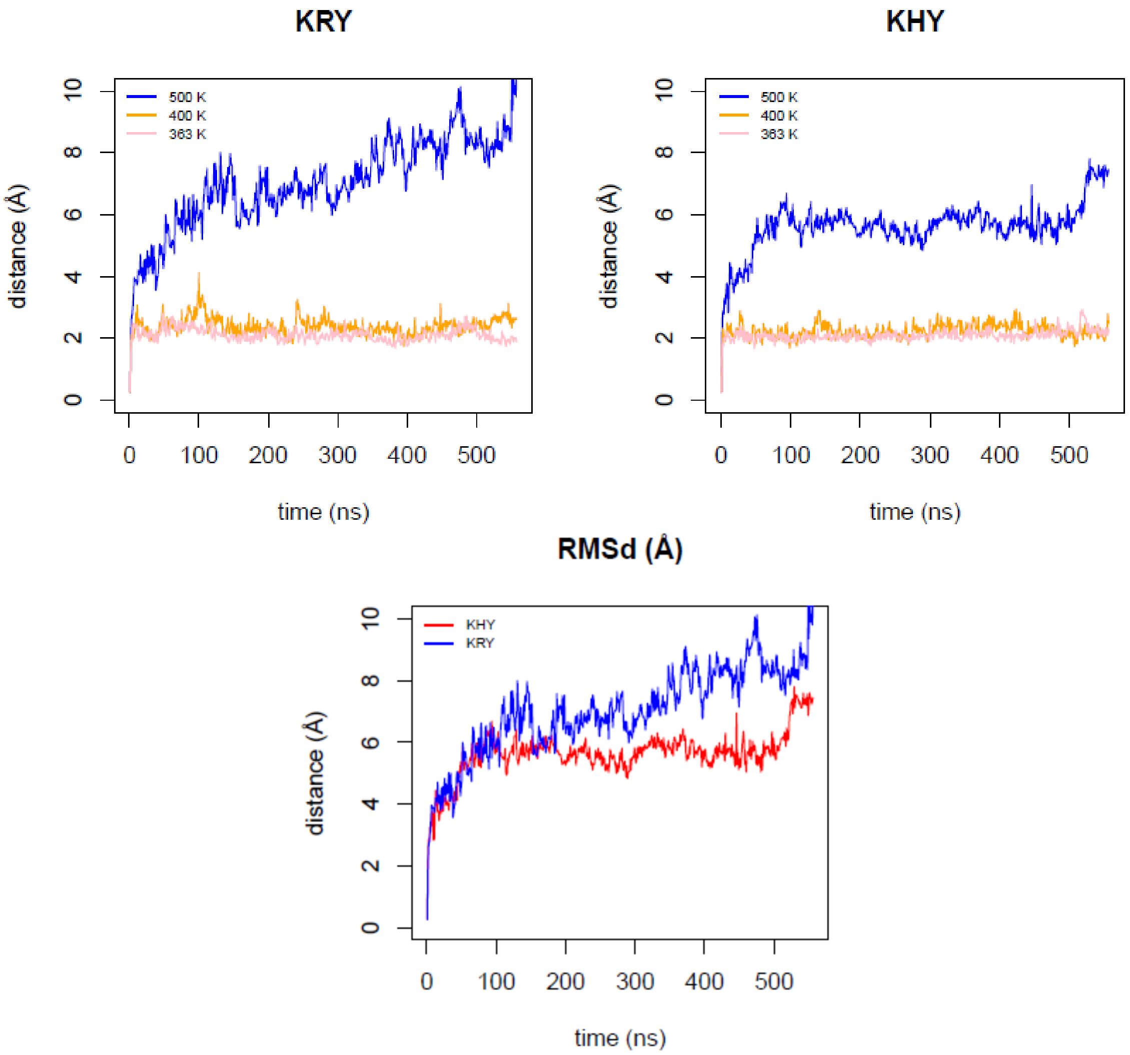

Figure S4. RMSd (Å) evolution of the two variants K166/H174/Y194 and K166/R174/Y194 along 0.5 μs of unbiased MD simulation.

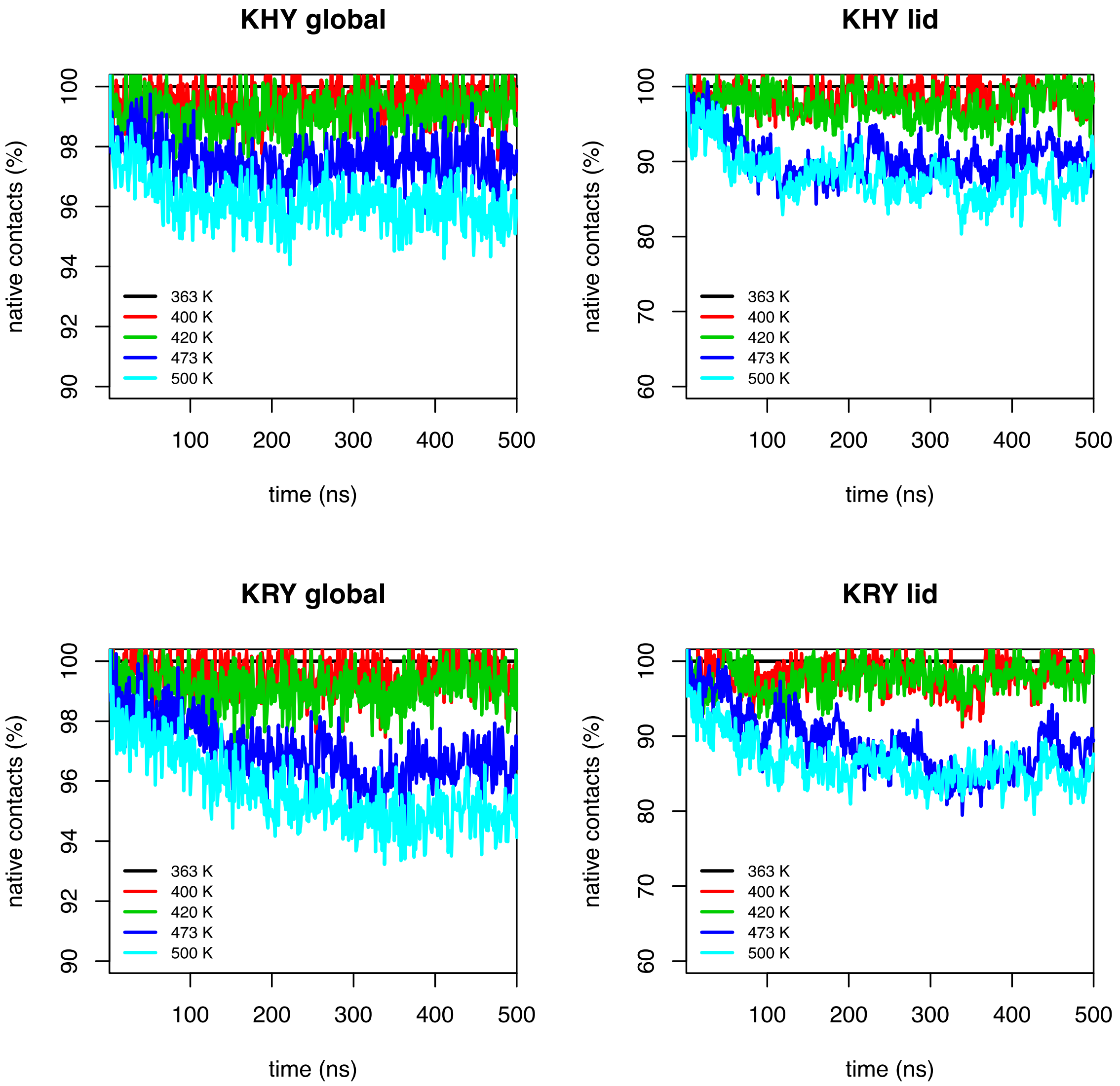

Figure S5. Native contacts (%) evolution of the two variants K166/H174/Y194 and K166/R174/Y194 along 0.5 μs of unbiased MD simulations at different temperatures. Together with the global number of native contacts, the evolution of the residues of the lid is also shown.
